# Influence of Oxygen Management on Color and Phenolics of Red Wines

**DOI:** 10.3390/molecules28010459

**Published:** 2023-01-03

**Authors:** Marioli Carrasco-Quiroz, Maria del Alamo-Sanza, Ana María Martínez-Gil, Rosario Sánchez-Gómez, Víctor Martínez-Martínez, Ignacio Nevares

**Affiliations:** 1 Department of Analytical Chemistry, UVaMOX—Universidad de Valladolid, 34004 Palencia, Spain; 2 Department of Agroforestry Engineering, UVaMOX—Universidad de Valladolid, 34004 Palencia, Spain; 3 Faculty of Science and Technology, Isabel I University, 09003 Burgos, Spain

**Keywords:** oxygen, oxygen consumption kinetics, oxygen saturation, phenols

## Abstract

Winemaking involves contact at different stages with atmospheric oxygen, the consumption of which determines its final properties. The chemical analysis of red wines subjected to consecutive cycles of air saturation has been extensively researched; however, the capacity to consume different doses of oxygen before bottling is an aspect that has been little studied. In this work, the effect of saturation of different levels of oxygen on the final characteristics of different wines made from Tempranillo and Garnacha grape extracts was studied. For this purpose, the wines were subjected to controlled oxygen saturation levels to simulate their possible oxygenation before bottling. The only difference was the phenolic composition of grape extracts that were reconstituted under the same conditions to avoid the interferences inherent to the fermentation process and the additives added in the winery. The kinetics of oxygen consumption was then evaluated and its effect on the color, antioxidant capacity, and phenols of three different wines was analyzed. This work shows the relationship between the oxidation state of wine and changes in its chemical composition. In addition, it provides insight into the effect of oxygen consumption before bottling on the properties of wines subjected to high and single doses of oxygen.

## 1. Introduction

The oxygen received by a wine during its production and storage plays a fundamental role in its final characteristics. Young wines have a high content of reactive oxygen species that trigger a series of chemical reactions influencing their attributes. High levels of oxygenation can have negative effects on wine, such as oxidation of phenolic and volatile compounds [1], leading to reduced or oxidized aromas [2,3,4,5] and off-flavors related to aldehydes and bacterial spoilage. However, for many years the effect of adding small amounts of oxygen to wine (micro-oxygenation) has been studied, demonstrating its influence on the chemical and sensory characteristics of wine such as increased olfactory intensity, structure, and complexity in the mouth [6].

Improvements in color stability, increase in color intensity [2,7,8,9,10,11,12], and degradation of anthocyanins [13] presenting a rather unstable chemical reactivity [14], generate more stable derivative pigments after wine aging due to a series of degradation and oxidation reactions [15]. In addition, copigmentation phenomena are generated that also influence color changes during wine aging [16,17,18]. The controlled use of oxygen can increase fruity and spicy flavors and also decrease herbaceous aromas and astringency [10,19,20,21]. Furthermore, oxygen has an important influence on yeasts during wine fermentation. Oxygen additions have been shown to impact non-Saccharomyces yeasts, influencing higher yield and survival rates during the anaerobic stage, and affecting wine flavor and aroma [22,23]. In the same way, excessive exposure to oxygen could cause fermentation stops, causing an alteration in the aromatic quality of the wines and in their chemical components [23].

Other studies show the complexity of the processes caused by the controlled addition of oxygen in wines, much of the reactions depending on the variety, grape, and wine characteristics [24,25,26,27,28,29]; phenolic profile [30]; aging conditions in barrels [31,32,33] and in bottles [34,35]. Different authors have exposed wine to saturation cycles with air, reaching different amounts of dissolved oxygen from 6 mg/L to 7 mg/L in the first saturation [36,37] or between 5 and 8 mg/L with three cycles [38,39] or between 27 and 50 mg/L with four saturation cycles [40]. Regarding the analysis of oxygen consumption, it has been generally observed that the consumption rate after the first saturation cycle is higher in subsequent cycles [41,42,43,44,45]. Oxidized compounds formed at the first saturation are most probably less likely to consume oxygen from a following saturation [36,41,46]. It has been found that after a saturation, wine undergoes a loss of monomeric and total anthocyanins [37,46] as well as the production of new polymeric pigments (10–15%) directly related to oxygen consumption [41]. This loss of anthocyanins affects the chromatic characteristics of wines due to the progressive loss of pigments reflected in an increase in lightness (L*) [47,48]. When four saturations of a wine are performed, several authors report an increase in hue and loss of color intensity (CI) [20,37,41].

The differences reported in the various studies make it necessary to expand the information on the ability of wines to take up and consume oxygen—as well as the effect on spectral characteristics, anthocyanins, and antioxidant capacity—all under controlled conditions using a single dosage with different oxygen saturation levels in similar wines that differ only in their phenolic content.

## 2. Results and Discussion

### 2.1. Kinetics of Oxygen Consumption in Saturated Ws at Different Oxygen Levels

The process of saturating samples with different levels of dissolved oxygen is complicated [49], since each sample must reach the setpoint level while ensuring that it is not oversaturated. The time required to achieve the set O_max_ depended on the type of W and the setpoint oxygen level at saturation, varying between 5 and 20 min. The oxygen levels reached were 125 hPa, 123 hPa, and 126 hPa for W-A, W-B, and W-C, respectively, when subjected to Sat-1, 170 hPa, 166 hPa; and 166 hPa to reach Sat-2 in W-A, W-B, and W-C, respectively; 241 hPa, 255 hPa, and 239 hPa to reach Sat-3 in W-A, W-B, and W-C, respectively; and finally 295 hPa, 276 hPa, and 287 hPa to reach Sat-4 in W-A, W-B, and W-C, respectively. In other words, the samples were taken to four different oxygen levels so that the second level meant an increase of 40% with respect to the first and for the third and fourth, levels of 96% and 130% of oxygen with respect to Sat-1, respectively. These results showed that there were no significant differences in the oxygen level (O_max_) reached due to the type of W coming from different red grapes for any of the saturation levels—i.e., the three Ws reach the same oxygen partial pressure when saturated to Sat-1, Sat-2, Sat-3, and Sat-4—although the TPI of W-C was approximately half that of W-A and W-B. However, it is important to note that when working with white, rosé, and red wines not all reach 100% air saturation (pO_2_/0.2095 × P_atm_). Thus, previous works have found that only 86%, 82%, and 81% are reached for white, rosé, and red wines, respectively—i.e., the difference between the theoretical oxygen partial pressure and that reached by the samples is significantly lower for white wines (24 hPa), followed by rosé (30 hPa), and red wines (33 hPa) [49].

**Figure 1:**
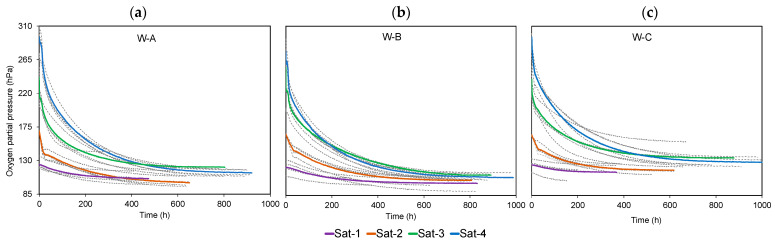
Oxygen consumption kinetics (**a**) W-A (*n* = 4), (**b**) W-B (*n* = 4), and (**c**) W-C (*n* = 4) for each saturation level (Sat-1: purple, Sat-2: orange, Sat-3: green, and Sat-4:blue).

Figure 1 shows the kinetics of consumption in quadruplicate of W-A and W-B of Tempranillo and W-C of Garnacha at each initial saturation level. The analysis of the curves, as described in the previous section, allowed us to establish the different parameters describing each of the kinetics. Table 1 presents the results of the multiple comparison ANOVA with Tukey’s test (*p* < 0.05) applied to the parameters describing the consumption kinetics in the different conditions for the three types of W. On an averaged basis in the case of W-A, it was found that when saturated with high oxygen contents (up to 296 hPa corresponding to Sat-4), the consumption rate was high and after 20 days (472 h) the dissolved oxygen level was the same as when saturated with Sat-3 up to 245 hPa (Figure 1a). That is, W-A samples with Sat-4 consumed 41 hPa (1.9 mg/L) more than W-A Sat-3 over the same time. At that moment, the values of cyanidin; peonidin; malvidin; antioxidant capacity; TPI; IC and the absorbances at 420, 520, and 620 were slightly higher in Sat-3 (non-significant differences), possibly because in that same time period they had consumed less oxygen. The W samples with Sat-4 ended the consumption kinetics with a lower dissolved oxygen level, 114 hPa (5.3 mg/L), than the samples that were saturated Sat-3, which ended with 121 hPa, (5.7 mg/L). This trend was also observed when the samples were brought to oxygen levels lower or equal to air: the samples saturated with air (Sat-2) and Sat-1 took 15 days (357 h) to reach the same DO level, consuming the first 42 hPa (2 mg/L) more than the saturated Sat-1. At the end of the consumption kinetics, the Sat-2 samples maintained lower levels reaching an Omin of 100 hPa (4.7 mg/L) compared to Sat-1 with an O_min_ of 105 hPa (4.9 mg/L). Chemical analyses performed at this time showed that the delphinidin, cyanidin, petunidin, peonidin, malvidin, TPI contents, and absorbances at different wavelengths of the spectrum between 330 nm and 650 nm were higher in the Sat-2 samples, being lower in antioxidant capacity and luminosity than in the Sat-1 samples. This trend was also found after 19 days (467 h) in the samples with Sat-3 and Sat-4 of W-C, which reached the same DO level (Figure 1c). In this case, the contents of cyanidin, petunidin, peonidin, DPPH, as well as the absorbances at different wavelengths of the spectrum between 330 nm and 650 nm were higher in the samples with Sat-3 oxygen level. This did not occur in the case of the comparative Sat-1 and Sat-2.

In the case of W-B, the above-mentioned behavior occurred after 7 days (185 h) of oxygen consumption in the case of the samples with Sat-3 and Sat-4 (Figure 1b), as opposed to the 20 days required in the case of W-A and W-C. At that time the delphinidin, cyanidin, petunidin, peonidin, malvidin, antioxidant capacity (ABTS), TPI, and absorbances at different wavelengths were found to be higher in the W-B samples with Sat-3; while the antioxidant capacity (DPPH), and the color parameters b*, a*, Cab*, and hab* were higher in the samples with Sat-4. These data tell us that the more available oxygen the samples have, the more oxygen they consume, causing the formation of new, more oxidizable compounds, such as aldehydes, mainly acetaldehyde which plays an important role in oxidation [50]. This was very evident in W-A, and not related to the initial content in total phenols, which was very similar in W-A and W-B (TPI of 34.8 and 34.5, respectively) and significantly lower than W-C (TPI of 16.3). This information corroborates what has been indicated in other studies [51,52] where both white and red wines were shown to be capable of consuming similar levels of oxygen. However, these works do not agree with what was observed by other researchers [53] who concluded that red wines could consume more oxygen than whites. Although one might think of the influence of the metals iron (Fe) and copper (Cu), as they participate in the interaction of polyphenols with oxygen [54,55], in the present work the content of these metals in the three Ws is the same. However, it did relate to changes in the level of phenols (start and end of oxygen consumption) since they presented a greater loss of TPI at the end of oxygen consumption in the case of the W-A samples with Sat-4 compared to the samples maintained in an inert atmosphere (Sat-0, anoxia).

**Figure 2:**
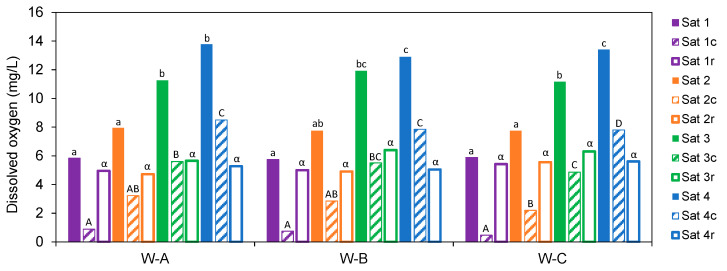
Dissolved oxygen level (mg/L) consumed (c) and remaining (r) (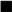: dosed oxygen; 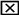 oxygen consumed; 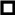: remaining oxygen) (Sat-1: purple, Sat-2: orange, Sat-3: green, and Sat-4:blue). For each wine, different letters indicate significant differences among different saturation levels (α < 0.05), lower case letters for the oxygen dose, capital letters for oxygen consumed and Greek letters for oxygen remaining level.

The analysis of the amount of oxygen consumed by the different samples indicates that, on average, all the samples consumed more oxygen as more was provided. Thus, when saturated at Sat-1 (5.8 mg/L), they consumed 12% of the dosed oxygen (0.7 mg/L); while, if saturated with 7.8 mg/L, they consumed 35% (2.76 mg/L), and when given a single dose of 11.5 mg/L, they were observed to consume 5.33 mg/L, 46% of the available oxygen, and at the highest dose of 13.4 mg/L they consumed 60% of that available (8 mg/L). Specifically, W-A consumed 0.89 mg/L, 3.22 mg/L, 5.61 mg/L, and 8.5 mg/L of oxygen after being subjected to Sat-1, Sat-2, Sat-3, and Sat-4, respectively; which meant a consumption of 15%, 41%, 50%, and 62% of all the oxygen available to them at each level. In the case of W-B the oxygen consumption was of the same order: 0.75 mg/L, 2.85 mg/L, 5.51 mg/L, and 7.85 mg/L after being subjected to the different saturation levels; which meant a consumption of 13%, 37%, 46%, and 61% of all the oxygen available at each level. In all the described cases of W-A and W-B—except in the Sat-4 samples—the oxygen consumption was significantly higher than that found for the W-C samples, with a consumption of 0.47 mg/L, 2.20 mg/L, 4.86 mg/L, and 7.80 mg/L of oxygen after being subjected to the different saturation levels, which meant a consumption of 8%, 28%, 44%, and 58% of all the oxygen available at each level (Figure 2).

These results indicate that when the samples were taken to an oxygen level below saturation with air (Sat-1), which could simulate a racking process at 16 °C, they did not consume all the available oxygen, leaving a very similar dissolved oxygen remnant in all the Ws (about 5.12 mg/L). The same occurred when the samples were taken to the Sat-2 level, in which a dissolved oxygen remnant of 5.06 mg/L was left, somewhat higher when taking them to the Sat-3 level (6.12 mg/L) and of the same order as those saturated at Sat-4 (6.12 mg/L). That is, when the samples were subjected to saturation levels with medium, high, and very high oxygen, they consumed significantly higher amounts according to the increase in available oxygen, but in all cases the remaining oxygen not consumed was very similar (Figure 2). Therefore, compounds other than phenolics play an important role in the depletion of available oxygen observed in commercial red wines [49].

**Figure 3:**
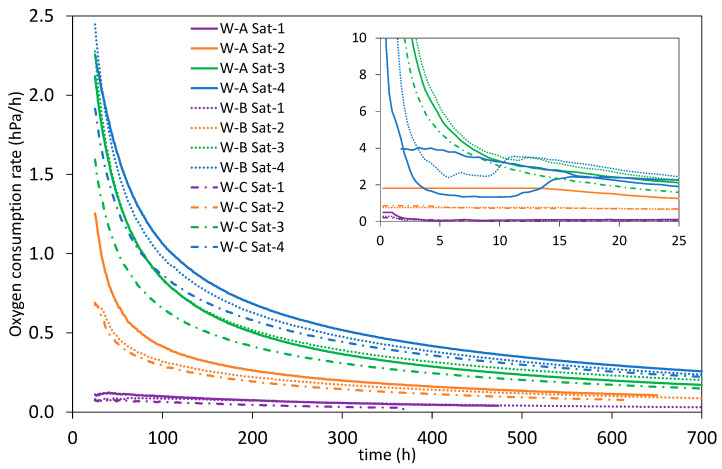
Oxygen consumption rate (Sat-1: purple, Sat-2: orange, Sat-3: green, and Sat-4:blue).

Analyzing the rate of oxygen consumption, it was found that it varied according to the type of W and the level of dissolved oxygen reached at saturation (O_max_) (Figure 3). The more oxygen the samples had available, the higher the rate of consumption of available oxygen until O_min_ was reached, this being especially relevant in the first few hours (zoom Figure 3). These results confirm what was observed by Picariello et al., 2020 [44], who described a rapid oxygen consumption over the first day after saturation, corroborating what was described by Ribéreau-Gayon, 1933 [56], who for the first time showed an initial rapid consumption attributed to Fe(II) oxidation and later corroborated by Danilewicz, 2013 [57].

Table 1b shows that for all three Ws the initial consumption rate was higher in the samples with higher oxygen levels (Sat-3 and Sat-4). When samples were saturated with oxygen levels equal to or lower than air, their consumption rate was significantly lower (Figure 3), with a maximum oxygen consumption of 2 hPa/h (0.1 mg/L·h) observed in W-A during the first 15 h. When samples were saturated with oxygen levels lower than what was present in air, oxygen was consumed very slowly (below 0.15 hPa/h in the first few hours, zoom Figure 3) and after 15 days the rate of oxygen consumption stabilized at 0.05 hPa/h (0.002 mg/L·h) in all samples analyzed, regardless of their nature. In the case of saturating the samples with the Sat-4 level, in the first 3 h the consumption rate dropped below 0.23 mg/L·h (5 hPa/h), while the samples with Sat-3 level needed twice as long (7 h) to reach the same consumption rate of 5 hPa/h (Figure 3).

When samples were saturated with oxygen levels lower than what was present in air, oxygen was consumed very slowly (below 0.15 hPa/h in the first few hours, zoom Figure 3) and after 15 days the rate of oxygen consumption stabilized at 0.05 hPa/h (0.002 mg/L·h) in all samples analyzed, regardless of their nature. In the case of saturating the samples with the Sat-4 level, in the first 3 h the consumption rate dropped below 0.23 mg/L·h (5 hPa/h), while the samples with Sat-3 level needed twice as long (7 h) to reach the same consumption rate of 5 hPa/h (Figure 3). It was observed that, after 15 h, oxygen consumption slowed down in all cases, and as can be seen in Figure 3, all samples maintained an oxygen consumption rate according to the saturation level, with the highest rate for those saturated with Sat-4, followed by Sat-3, Sat-2, and finally Sat-1 until the consumption kinetics ended and Omin was reached. Similar behavior was observed with the parameters related to the area under the consumption curve, with a greater area under the curve, the higher the level of oxygen in saturation, as reflected in the three parameters A_max_min_, AO_max_min_, and AO_90_10_, which presented statistically significant differences for all Ws with Sat-1 and Sat-4. However, no statistically significant differences were observed in the minimum residual oxygen level (O_min_) attained by the W samples with each of the different saturation levels reached, but yes in the time required for the samples to reach that level of dissolved oxygen (t_O_min_) in W-B and W-C when subjected to Sat-1 and Sat-4 (Table 1b).

These results indicated that the samples consumed all the available oxygen quickly, developing more reactions with oxygen by generating highly oxidizable compounds. This may be due to the reactions that occur once oxygen is reduced to hydrogen peroxide [58] with the Fenton reaction [59]. This peroxide can react with ferrous or cuprous ions and form hydroxyl radicals capable of oxidizing ethanol to acetaldehyde [54,60], which reacts with numerous compounds, such as anthocyanins generating more stable and complex phenolic compounds [61]. However, in the case of having low oxygen levels, the oxidation products are less oxidizable and thus the rate of consumption is reduced more rapidly, as seen in the W-C samples with Sat-3 and Sat-4. Since W-C had a lower TPI content, this behavior may be due to the lower content of phenolic compounds which are the primary substrates for oxidation [26,58,62,63]. These results have been described by Picariello et al., 2017 [64], showing a direct connection between the anthocyanin/tannin ratio, red wine oxygenation, and the reactions occurring in the wine. The present work was developed under the conditions of an aging room (16 ± 0.2 °C), which influenced the rate of oxygen consumption. Moreover, as described in the first section, although initially the samples subjected to the four saturation levels reached different levels of dissolved oxygen (O_max_), it was found that after 24 h consuming oxygen at different rates, all of them reached a rate below 2.5 hPa/h. The consumption rate is affected by the temperature at which the consumption kinetics develops, in this case at 16 °C, so when the kinetics develops at 35 °C, the rate is between 3 and 6 hPa/h after saturation with air [65]; while at Sat-2, corresponding to a saturation of 7.8 mg/L O_2_ (similar saturation to air), the rate is between 1 and 2 hPa/h. Therefore, regardless of the type of sample analyzed, at 16 °C oxygen consumption and thus the evolution caused by high oxygen levels slows down.

Grape variety plays a very important role in the oxidability of wines, [66]. It has been shown that the rate of oxygen consumption depends on wine composition [67], who found a high correlation (r = 0.959) between antioxidant capacity and total phenol content (between 1018 and 3545 mg/L in reds, and between 262 and 1425 mg/L in whites, expressed as gallic acid). These results have been recently corroborated by Hernández et al., 2021 [68], with a positive correlation between antioxidant capacity and flavanol composition, monomer content, and shorter proanthocyanidins. In the present work, it is important to remember that the only difference between the Ws is the phenolic content, since the three grape extracts were reconstituted in the same way, which avoided the interferences inherent to the fermentation process and the additives added in the winery. As shown in Table 1, the samples of the Garnacha variety (W-C) consumed oxygen faster (they had a higher consumption rate, R_max_) although they consumed less oxygen (∆O_max_min_) presenting higher levels of residual oxygen (O_min_), possibly due to their lower phenol content. Marrufo-Curtido et al., 2018 [69] described a higher “explosive stage” of oxygen consumption, the higher the Cu/Fe ratio, absorbance at 520 nm and guaiacylpyranopeonidin-3-O-glucoside content, while acetaldehyde content produced a negative effect on the “explosive” rate. These results were similar to those observed in the present work (Table 1b), where the consumption rate of the first 10% of the total oxygen consumed (V_cons10%_), was higher in the samples with a higher amount of available oxygen (Sat-3 and Sat-4). Those authors indicated that oxygen consumption tends to decrease with successive oxygen saturations; however, in the present work when all the oxygen was dosed at once, the wines consumed more oxygen (parameter ∆O_max_min_, Table 1a)—in other words with a single dose of oxygen.

### 2.2. Effect of Oxygen Consumption on Phenol Composition and Color

**Figure 4:**
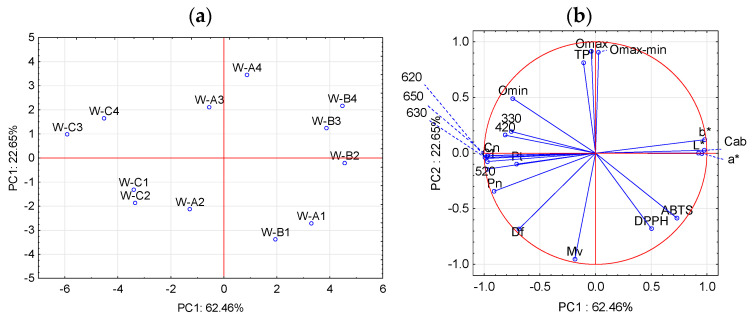
Principal component analysis (PCA) for W-A, W-B, and W-C presents distribution of the samples (**a**) and of the variables (**b**) (Legend in Table 2).

The effect of the different amounts of oxygen consumed on the properties of the wine was studied by evaluating the difference between the characteristics of the samples after consuming the oxygen reached at Sat-1, Sat-2, Sat-3, and Sat-4 versus the characteristics of the same samples stored under anoxic conditions (Sat-0). These differences may be attributable to the amounts of oxygen consumed rather than to changes occurring over time. Therefore, the oxygen consumed after dosing the four levels described above (∆O_max_min_) and the differences occurring in different chemical and color parameters were correlated for each type of W (Table 2). Figure 4 presents the results obtained by PCA analysis of these data, the distribution of the samples (a) and of the variables (b) in the plane formed by PC1 and PC2. As can be seen in Figure 4b, PC1 explains 62.46% of the variability of the data and is positively related to the color parameters, L*, a*, b* Cab, and to the antioxidant capacity, ABTS and DPPH (Figure 4b) close to the W-B samples (Figure 4a). On the other hand, 330 nm, 420 nm, 520 nm, anthocyanin content, and minimum oxygen (O_min_) are negatively related to PC1 (Figure 4b) close to samples W-C (Figure 4a). PC2 explains 22.65% of the variability of the data and differentiates samples with oxygen contents Sat-1 and Sat-2 (Figure 4a) defined by better maintaining malvidin content (Figure 4b), while samples with oxygen levels above air Sat-3 and Sat-4 (located on the positive axis PC2, Figure 4a) that consume higher amount of oxygen and present higher level of TPI (4b).

**Figure 5:**
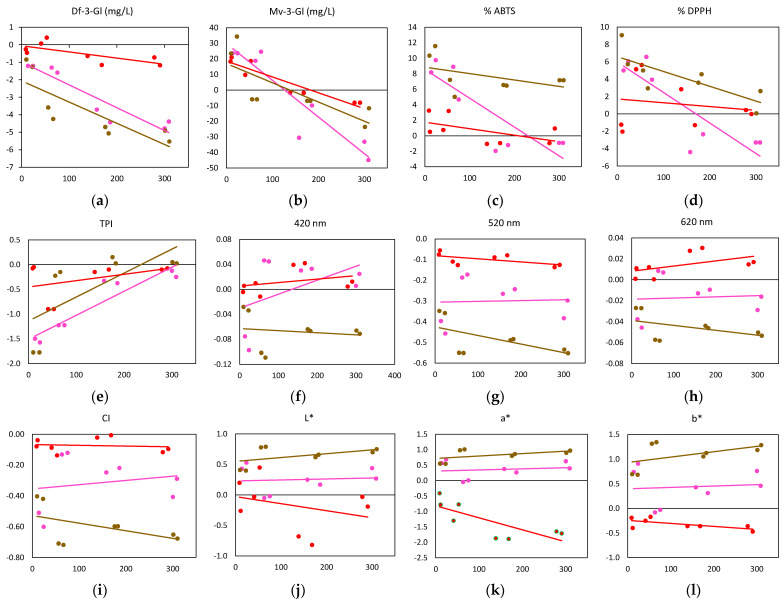
Oxygen consumed (hPa, *X*-axis) and the change in each parameter compared to the inert condition (Sat-0), *Y*-axis for W-A (pink), W-B (brown), and W-C (red) for different time (days, *Y*-axis). (**a**) Df-3-Gl: delphinidin-3-O-glucoside; (**b**): Mv-3-Gl: malvidin-3-O-glucoside; (**c**) %ABTS: Antioxidant capacity; (**d**) %DPPH: Antioxidant capacity; (**e**) TPI: Total Phenols Index; (**f**) 420 nm: absorbances at 420 nm; (**g**) 520 nm: absorbances at 520 nm; (**h**) 620 nm: absorbances at 420 nm; (**i**) CI: Color Intensity; (**j**) L*: black to white lightness; (**k**) a*: red to green; (**l**) b*: blue to yellow.

Table 2 shows that as the oxygen consumed (∆O_max_min_) increased, there was a significant loss of anthocyanins, especially malvidin (correlation of −0.908, −0.819, and −0.950 with W-A, W-B and W-C, respectively) and delphinidin (correlation of −0.922 and −0.900 with W-A and W-B, respectively) compared to the same samples maintained under anoxic conditions during the same period of time. Samples W-C, having a lower initial content, showed lower losses of these anthocyanins, followed by W-A and W-B. Figure 5a represents the average oxygen content consumed by each W in each saturation situation (Sat 1, 2, 3, or 4) versus the difference in the average delphinidin content after consuming that amount of oxygen and the content in the sample in the absence of oxygen (Sat-0). It is observed that all samples lost more delphinidin when saturated with different levels of oxygen than when kept free of oxygen, and that samples W-A and W-B showed the greatest loss as oxygen consumption increased. As for malvidin (Figure 5b), it can be seen that after consuming amounts of oxygen below air (Sat-1 and Sat-2) the samples maintained a higher content of this anthocyanin than in the absence of oxygen, as shown in Figure 4a. However, when the samples were subjected to high doses of oxygen (Sat-3 and Sat-4), they lost more malvidin than if they were in anoxia, this tendency being more marked in W-A. Initially, W-A and W-B had a higher TPI content showing significant correlation with the oxygen consumed—0.932 and 0.791, respectively—indicating the formation of new compounds with oxygen intervention (Figure 5c). These reactions gave rise to compounds with a lower antioxidant capacity (%DPPH), significant in the samples with higher levels of phenolic compounds (−0.853 and −0.786 for W-A and W-B). Thus, these samples presented a higher antioxidant capacity (%ABTS and %DPPH) with oxygen dosages below or equal to air (Sat-1 and Sat-2), compared to samples kept in anoxic conditions. On increasing the oxygen dosage (Sat-3 and Sat-4), the antioxidant capacity decreased (Figure 5d,e), being lower than in the oxygen-free samples.

**Figure 6:**
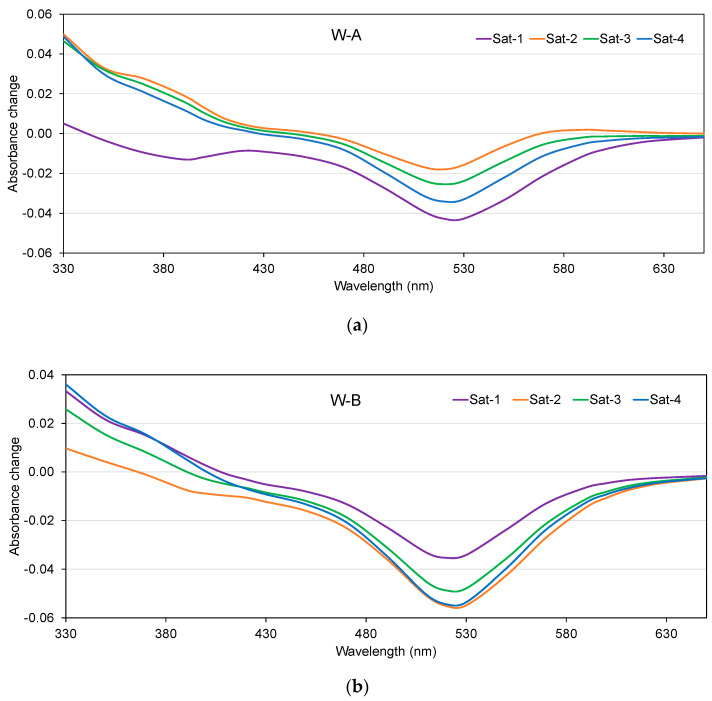

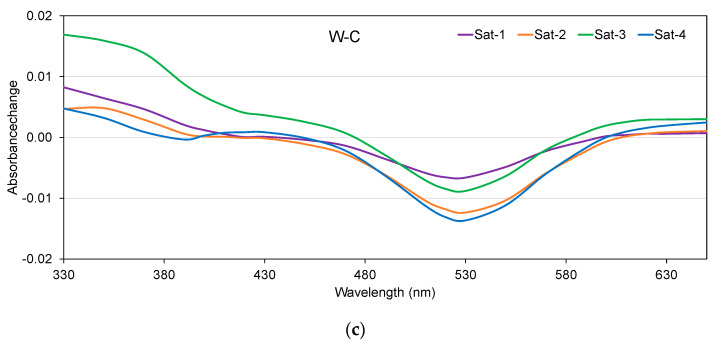
Changes in the spectrum of the Ws with different levels of oxygen (Sat-1: purple, Sat-2: orange, Sat-3: green and Sat-4: blue) with respect to the changes that occurred in Sat-0 (inert atmosphere). (**a**) W-A, (**b**) W-B, and (**c**) W-C.

However, when the Ws were subjected to Sat-1 they showed the lowest absorbance losses at 520 nm (0.0354). For the samples with lower phenolic content, W-C, it was found that the highest loss of absorbance (0.014) occurred with high doses of oxygen (Sat-4), but very close to those obtained with Sat-2 and Sat-3, with losses of 0.012 and 0.009. It was found that the W-C samples presented higher absorbance than the oxygen-free samples (Figure 6) in the 600–650 nm range after consuming oxygen, which may be related to the formation of portisins, a type of anthocyanin vinyl flavanol pigment of a blue color [15]. This result is of great interest, as it tells us that—for the same conditions of Sat-1, Sat-2, Sat-3, and Sat-4—samples W-C maintained or increased the level of violet hues (Figure 5i), while samples W-A and B maintained or lost these hues.

As for the color parameters, the results of the color intensity indicated that with the increase in oxygen consumption the samples showed lower CI (Figure 5f) in all cases, especially W-A and W-B. The same tendency was found in the case of measurements at 520 nm (Figure 5g) which reflect the loss of red color, typical of an aging process [70]. When measured at 420 nm (Figure 5h), which represents brownish tones, it can be observed that with oxygen consumption, samples W-A and W-C showed levels similar to those of samples stored in anoxia. The increase or decrease in absorbance between 330 and 650 nm caused by oxygen consumption for each W at different saturation levels (Sat-1, Sat-2, Sat-3, and Sat-4) with respect to anoxic conditions (Sat-0), is shown in Figure 6. The range of absorbance between 370 and 470 nm, which is related to yellow-orange tones, and the range from 520 to 670 nm related to red-purple colors is interesting. Samples after consuming oxygen showed a higher absorbance than inert samples between 330 and 370 nm, indicating an increase in yellow-orange shades. Absorbances near 310–330 nm are characteristic of acylation with p-coumaric acid [71] and are also related to compounds such as pyranoanthocyanins [72], typical of an aged wine. Thus, W-A samples with oxygen saturation at Sat-2, Sat-3, and Sat-4 levels showed the largest increases of about 0.035 in absorbance, while they presented a slight loss of absorbance (close to 0.003) with small amounts of oxygen (Sat-1). In the case of samples W-B and W-C, no trends according to oxygen consumption were observed in this range of absorbance. Between the absorbances at 420 and 440 nm, the smallest variations were observed with respect to the oxygen-free samples, although at 440 nm a loss of absorbance began to be appreciated in the three Ws. These changes may be due to the interaction between catechins and anthocyanins for the formation of new yellowish substances responsible for the absorbance around 440 nm [73]. Absorbances near 520 nm are related to the form of the bright red flavilium cation, which may be present in the form of colorless carbinol pseudobase, in the purple quinoidal form, in addition to vitisin A (a type of pyranoanthocyanin with a maximum absorbance at 512 nm) and malvidin-3-glucoside with a maximum absorbance of 524 nm [15]. The absorbance at 538 nm is related to a pyranoanthocyanin-vinylphenol pigment with a more purple hue in acid solution [74] and at 575 nm to blue pigments with a pyranoanthocyanin structure linked to a flavanol by a vinyl bridge [75]. The study of this area of the spectrum shows the greatest differences according to the oxygen dose (Figure 6).

Thus, in the case of the W-A samples, the greatest losses in absorbance at 520 nm were found when the samples were subjected to Sat-1, with a loss of approximately 0.043, which corresponds to 58% more than what was observed in the samples with Sat-2 (loss of 0.018), followed by Sat-4 and Sat-3, which lost 0.034 and 0.025. The W-B samples showed the highest absorbance losses at 520 nm due to oxygen consumption, 0.055 and 0.054 in the samples with Sat-2 and Sat-4, respectively, followed by Sat-3 with −0.049.

The W-C samples of the Garnacha variety, with a lower phenolic content showed a significant correlation between oxygen consumption and a* (−0.813) and Cab* (−0.820) reflecting a loss of color with oxygen consumption (Table 2). Other authors—such as Sánchez-Gómez et al.,2020 [12], Cejudo-Bustamante et al., 2011 [21], Atanasova et al., 2002 [76] and Picariello et al., 2020 [44]—showed that oxidation processes facilitate an increase in CI in samples subjected to micro-oxygenation processes, i.e., very low oxygen dosage compared to studies in which wines are saturated with air, or subjected to several aeration cycles or, as in this work, when wines are saturated all at once with higher amounts of oxygen than aeration. In Figure 5, a decrease in a* can be observed in the W-C samples as oxygen consumption increased, indicating that red hues were lost, similarly to what occurs in an aging process of red wines due to the increase in the degree of polymerization and copigmentation in phenolic compounds [21,77,78]. However, in samples with a higher phenolic content, W-A and W-B oxygen consumption implied maintaining or increasing a* with respect to samples subjected to anoxia. Very similar results are shown for parameter b* (Figure 5k).

These results indicate a color modification with oxygen consumption: red tones are replaced by yellow-orange tones, due to the oxidation of phenols and the formation of stable pyroanthocyanins that present orange tones [79,80]. In general, it was observed that the higher the phenol content (W-A and W-B), the higher the oxygen consumption causing a higher gain in L*, a*, and b* with respect to samples stored in the absence of oxygen.

## 3. Materials and Methods

### 3.1. Grape Extracts (GEs)

Grape extracts (GEs) were prepared using grape varieties harvested in 2017 (two with the Tempranillo variety (GE-A and GE-B) from Bodegas Ramón Bilbao (Haro, La Rioja, Spain) and one Garnacha (GE-C) from Bodegas y Viñedos Ilurce (Alfaro, La Rioja, Spain), all supplied by the Laboratorio de Análisis de Aromas y Enología (LAAE) of the Universidad de Zaragoza (Zaragoza, Spain), obtained by the method indicated in Alegre et al., 2020 [81]. The extracts were prepared at LAAE following their method published in 2020 and were kept frozen prior to shipment to UVaMOX. They were stored frozen in the UVAMOX laboratory until the preparation and analysis of grape extract wines (Section 3.2) in 2019.

Briefly, 10 kg of grapes were destemmed and crushed in the presence of 15% (v/v) ethanol and 5 g/hL potassium metabisulfite (Merck, Darmstadt, Germany), macerated for 7 days at 13 °C, then pressed, filtered, and stored at 5 °C in the dark. This ethanolic must was dealcoholized in a rotary evaporator system (Buchi R-215 equipped with a Buchi V-700 vacuum pump, Flawil, Switzerland) which was then passed through a prepared 10 g C18 cartridge previously conditioned with 44 mL methanol and 44 mL milli-Q water with 2% ethanol. The cartridges were then washed with 88 mL of milli-Q water at pH 3.5 and dried by passing air through them. Finally, grape extracts (GEs) were recovered by elution with 100 mL ethanol.

### 3.2. Grape Extract Wines (Ws)

Grape extracts were reconstituted to simulate a wine (W) under these conditions: pH 3.3; 1 mg/L Fe^2+^; 0.1 mg/L Cu^2+^; 1 mg/L Mn^2+^; 12% (v/v) alcoholic strength, 10 mg/L acetaldehyde and 5 g/L total acidity as tartaric acid according to the method of Carrasco-Quiroz et al., 2022 [65]. Pure iron (II) chloride 4 hydrate, copper (II) chloride 2 hydrate, manganese (II) chloride 4 hydrate (PanReac-AppliChem, Barcelona, Spain), acetaldehyde (>99.9%, Fluka CHECK), L (+)-tartaric acid (Scharlab, S.L., Barcelona, Spain), and sodium hydroxide solution (Labbox Labware, SL, Barcelona, Spain) were used.

#### 3.2.1. Saturation at Different Oxygen Levels

Five oxygen levels, including an oxygen-free one, were established to saturate the Ws, which were tempered at 16 ± 0.2 °C (temperature of a barrel room) for 24 h prior to saturation. Each saturation was performed using a ceramic microdiffuser at a flow rate of less than 1 mL/min, following the procedure established by Näykki et al., 2013 [82]. Nitrogen, oxygen, and argon (Carburos Metálicos, Air Products Group, Barcelona, Spain) were used, dosing the necessary amounts to reach each of the levels with a Gm-3 gas mixer (Sensor Sense, Nijmegen, The Netherlands). To maintain the samples without oxygen, Sat-0 was saturated with argon, working at a Patm of 937 hPa, for Sat-1 125 hPa (5.8 mg/L) was reached on average, for Sat-2 167 hPa (7.8 mg/L), in the case of Sat-3 245 hPa (11.5 mg/L) and finally for Sat-4 the samples were taken to 286 hPa (13.4 mg/L) with mixtures of nitrogen and oxygen.

#### 3.2.2. Oxygen Consumption Measurement through Kinetics and Kinetic Curve Data Processing

After saturating the samples with different oxygen levels (Sat 1-2-3-4), the dissolved oxygen content was monitored to determine the kinetics of oxygen consumption.

The oxygen consumption kinetics of each W was performed in quadruplicate for each of the oxygen saturation levels, i.e., 48 kinetics were performed in total (3 Ws × 4 saturations × 4 repetitions), collecting the measurement of available dissolved oxygen every 15 min. This was not collected in the case of the level corresponding to 0% oxygen—anoxia—(Sat-0) as there was no oxygen to consume. The measurement of dissolved oxygen (DO) evolution was carried out with a SensorDish SDR multi-reader device (PreSens Precision Sensing GmbH, Regensburg, Germany) equipped with hermetically sealed 3 mL glass vials with precision valve screw caps (Restek Innovative Chromatography Products, Bellefonte, USA). Each vial had an isolated optical oxygen sensor SV-PSt5 (PreSens Precision Sensing GmbH, Regensburg, Germany) integrated into the bottom. The oxygen sensors in each vial were calibrated according to the manufacturer’s protocol, taking into account the measurement temperature 16 ± 0.2 °C, with measurements taken at two calibration points: oxygen-free water at a concentration of 0 mg/L (0% air saturation) and saturated air (100% air saturation). The 48 kinetics were processed following the method established by del Álamo-Sanza et al., 2021 [49] obtaining the characteristic parameters of the curves describing each kinetic. The consumption curve data analyzed were those found from the maximum oxygen level reached by each W to the minimum value, considering the minimum oxygen value that recorded after performing several stable measurements: this moment was considered the end of the oxygen consumption kinetics. Subsequently, the replicates of the same sample were combined to obtain two representative curves: one representing the data obtained from the mean minus the standard deviation and another curve representing the data obtained from the mean plus the standard deviation of the four replicates of each sample. The parameters selected were: maximum/initial oxygen (hPa) = O_max_; minimum/residual oxygen (hPa) = O_min_; total oxygen consumed (hPa) = ∆O_max_min_; oxygen when half of the total consumption time (hPa) had passed = O_int_; oxygen representing 90% of the range between the maximum and minimum values—i.e., when the first 10% of total oxygen (hPa) had been consumed = O_90_; variation between 90% and 10% oxygen (hPa) = ∆O_90_10_; area under the oxygen consumption curve (hPa) = A_max_min_; area under the oxygen consumption curve between maximum and minimum oxygen (hPa) = AO_max_min_; variation between 90% and 10% of oxygen consumed (hPa) = ∆O_90_10_; time in hours it took to consume all the oxygen (h) = t_Omin_; time it took to reach O_90_ = t_0_90_; time it took to reach the minimum oxygen consumption rate (h) = t_R_min_; rate of oxygen consumption (hPa/h) = R_max_; the area divided by the time it took to consume = R_mean_; rate of consumption of ∆O_max_min_ (hPa/h) = V_cons_; and rate of consumption to consume the first 10% of oxygen consumed (O_90_) (hPa/h) = V_cons10%_.

### 3.3. Chemical Analyses

These samples were kept in a cellar cabinet at a constant temperature of 16 ± 0.2 °C and in darkness (Liebherr Vinoteck, Owen, Germany) during the oxygen consumption process. The analyses of the Ws were performed in duplicate, first after reconstitution and then after the end of the kinetics of oxygen consumption dosed at four levels, at which time the samples maintained in anoxia were also analyzed.

#### 3.3.1. Spectra, Color Parameters, and Total Phenols Index

Visible spectra in the range 330–650 nm at 5 nm intervals were obtained using a PerkinElmer LAMBDA 25 UV–vis spectrophotometer (Waltham, MA, USA). Quartz cuvettes of 1 mm thickness were used. Color analysis was performed by measurements at 420 nm, 520 nm, and 620 nm to calculate color intensity (CI) as the sum of these absorbances as defined by Glories, 1984 [83]. The CIELab parameters were calculated using the “OIV-MA-AS2-11 Method: Determination of chromatic characteristics according to CIELab” [84] (OIV, 2006). These parameters were: L*, describing black to white lightness; b*, blue to yellow; a*, red to green; C*, chroma or saturation; and H*, slant angle. The total polyphenol index (TPI) was analyzed by measuring the absorbance of the sample (1:25 pre-dilution) at 280 nm in a 10 mm quartz cell using the Ribereau-Gayon, 1970 [85] method.

#### 3.3.2. Antioxidant Capacity

Antioxidant capacity was measured by ABTS and DPPH. For the former, the Re et al., 1999 [86] method was followed with modifications. Briefly, the ABTS radical cation (ABTS+) was prepared by reacting a solution of ABTS (2,29-azinobis(3-ethylbenzothiazoline-6-sulfonic acid) di ammonium (Sigma Aldrich, Steinheim, Germany) 7 mM with 2.45 mM potassium persulfate (di-potassium disulfate, Sigma Aldrich, Steinheim, Germany). Then 1.95 mL of ABTS+ solution was added to 50 µL of the diluted sample (1:25) in a 1 cm thick cuvette and incubated at 35 ± 0.2 °C for 50 min before measuring at 734 nm. For DPPH analysis, the Brand-Williams et al., 1995 [87] method, with modifications, was chosen. Briefly, a solution of DPPH (2,2-Diphenyl-l-picrylhydrazyl, Sigma Aldrich, Steinheim, Germany) in ethanol (AGR ACS ISO absolute ethanol, Labbox Labware, Vilassar de Dalt, Barcelona, Spain) 6 × 10^−2^ mM was prepared. Then 1.95 mL of diluted DPPH solution was added to 50 µL of the diluted sample (1:25) in a 1 cm thick cuvette and incubated at 25 ± 0.2 °C for 60 min before measuring at 515 nm. Distilled water was used for the blank and Trolox (6-hydroxy-2,5,7,8-tetramethychroman-2-carboxylic acid, Sigma Aldrich, Steinheim, Germany) was used as the antioxidant standard. A concentrated solution of Trolox (30 mM) in ethanol (AGR ACS ISO absolute ethanol, Labbox Labware, Vilassar de Dalt, Barcelona, Spain) was prepared, and the corresponding calibration was performed at five points (between 0.05 and 1 mM) to construct a calibration curve obtaining regression coefficients between 0.98 and 0.99. In all cases, the samples, the blank and Trolox, were analyzed in duplicate.

#### 3.3.3. Analysis of Individual Anthocyanins

The analysis of the five main anthocyanins in the wines, delphinidin-3-O-glucoside (Df-3-Gl), cyanidin-3-O-glucoside (Cy-3-Gl), petunidin-3-O-glucoside (Pt-3-Gl), peonidin-3-O-glucoside (Pn-3-Gl), and malvidin-3-O-glucoside (Mv-3-Gl) was performed following the method described by del Álamo Sanza et al., 2004 [13]. The chromatographic separation was performed on a Fortis C18 column (with a particle size of 5 µm, a length of 250 mm and a diameter of 4.6 mm) (Sugelabor, Spain). The phases used were A: formic acid/H_2_O (15:85, v/v); B: formic acid/methanol/H_2_O (10:45:45, v/v); C: methanol/H_2_O (90:10 v/v). Anthocyanins were eluted using a flow rate gradient of 0.8 mL/min of solvents A, B, and C, with a colu temperature of 30 °C. The volume of sample injected was 40 µL. A scan was performed between 220 and 740 nm, with quantification at 528 nm being predominant. Anthocyanins were identified by comparing their spectra and retention times according to the method described above. Quantitative analysis was performed using the external standard method based on malvidin-3-O-glucoside (Mv-3-Gl), since it is the most representative anthocyanin in wines. The analysis of anthocyanins in each sample was performed in duplicate.

### 3.4. Statistical Analysis

Data analysis was performed with analysis of variance (ANOVA) according to Tukey’s test, with a significance level of 0.05 (α = 0.05) with the SPSS software (IBM SPSS Statistics 26 Portable License, USA) for Windows, and PCA analysis was performed by Statistica v12 (StatSoft GmgH, Hamburg, Germany).

## 4. Conclusions

The levels of dissolved oxygen reached by wines reconstituted from phenolic extracts of Tempranillo and Garnacha grapes were similar at each of the saturation levels studied, although their capacity to consume oxygen depended on their composition. Exposing the wines to single doses of oxygen higher than those achieved with aeration causes changes in their chemical composition characteristic of accelerated aging. The results indicate that the more oxygen the wines have available, the more oxygen they consume; however, the remaining oxygen in all cases is very similar and independent of the oxygen consumed. This aspect should be studied in depth to determine the mechanisms that define the level of remaining oxygen. These results highlight the opportunity to continue studying the aging capacity of a future wine from must, which will have an impact on the optimal treatment during its vinification process, allowing the future wine to express its maximum potential. Thus, there is a need to develop devices to evaluate the oxygen consumption capacity in routine analysis.

## Figures and Tables

**Table 1:** (**a**) W-A, W-B, and W-C parameters at the four oxygen saturation levels (Sat-1, Sat-2, Sat-3, and Sat-4). (**b**) W-A, W-B, and W-C parameters at the four oxygen saturation levels (Sat-1, Sat-2, Sat-3, and Sat-4).

(a)								
Saturation Level	O_max_	O_min_	∆O_max_min_	O_int_	O_90_	∆O_90_10_	A_max_min_	AO_max_min_
W-A								
Sat-1	125 ± 7.64 a	106 ± 7.59 a	18.9 ± 5.12 a	115 ± 7.17 a	123 ± 7.60 a	14.8 ± 4.20 a	52,544 ± 3208 a	2430 ± 763 a
Sat-2	170 ± 0.00 a	100 ± 6.08 a	69.3 ± 6.08 ab	135 ± 3.04 ab	162 ± 0.97 a	54.8 ± 4.57 ab	73,727 ± 3333 a	8488 ± 964 ab
Sat-3	241 ± 12.9 b	121 ± 14.0 a	120 ± 12.7 b	181 ± 11.9 bc	227 ± 11.7 b	94.0 ± 9.46 b	108,585 ± 7047 b	13,841 ± 2922 b
Sat-4	296 ± 15.7 b	113 ± 4.42 a	183 ± 13.2 c	205 ± 9.50 c	276 ± 13.9 b	144 ± 9.27 c	129,133 ± 4683 b	24,753 ± 1237 c
**W-B**								
Sat-1	123 ± 8.92 a	101 ± 8.93 a	16.5 ± 6.43 a	112 ± 6.91 a	120 ± 8.34 a	12.8 ± 5.16 a	87,881 ± 5116 a	2111 ± 1171 a
Sat-2	166 ± 0.00 ab	105 ± 5.24 a	61.3 ± 5.24 ab	136 ± 2.62 ab	160 ± 0.50 ab	48.6 ± 4.06 ab	93,639 ± 4316 ab	8882 ± 1211 ab
Sat-3	229 ± 22.0 bc	111 ± 3.45 a	118 ± 19.7 bc	170 ± 12.3 bc	215 ± 17.0 bc	92.1 ± 12.5 bc	117,120 ± 4988 bc	18,030 ± 2315 bc
Sat-4	277 ± 12.0 c	109 ± 4.39 a	169 ± 8.08 c	193 ± 8.07 c	260 ± 11.1 c	134 ± 6.40 c	129,846 ± 5545 c	22,466 ± 1490 c
**W-C**								
Sat-1	126 ± 7.67 a	116 ± 8.62 a	10.1 ± 1.19 a	121 ± 8.14 a	125 ± 7.79 a	7.70 ± 1.02 a	43,575 ± 3335 a	1004 ± 319 a
Sat-2	166 ± 0.00 a	119 ± 5.89 a	47.1 ± 5.89 b	142 ± 2.94 ab	161 ± 0.56 a	37.4 ± 4.66 b	78,183 ± 2677 a	4979 ± 1272 ab
Sat-3	240 ± 12.8 b	135 ± 14.8 a	105 ± 2.07 c	187 ± 13.8 bc	228 ± 13.4 b	82.7 ± 1.49 c	130,410 ± 13,035 b	12,003 ± 1541 b
Sat-4	296 ± 7.81 c	129 ± 5.90 a	167 ± 5.56 d	213 ± 6.34 c	278 ± 8.53 b	133 ± 5.45 d	161,098 ± 8410 b	22,971 ± 2900 c
**(b)**								
Saturation Level	A0_90_10_	t_Omin_	t_O_90_	t_R_min_	R_max_	R_medio_	V_cons_	V_cons10%_
**W-A**								
Sat-1	1667 ± 411 a	434 ± 34.4 a	17.6 ± 4.46 b	254 ± 97.6 a	0.91 ± 0.26 a	254 ± 138 a	0.04 ± 0.01 a	0.12 ± 0.01 a
Sat-2	5095 ± 703 ab	639 ± 7.78 a	4.19 ± 0.58 a	19.3 ± 6.78 a	19.3 ± 0.43 a	19.3 ± 9.58 a	0.11 ± 0.01 ab	1.81 ± 0.47 a
Sat-3	6808 ± 3511 ab	620 ± 232 a	0.16 ± 0.00 a	2.39 ± 0.50 a	90.2 ± 15.6 b	2.39 ± 0.71 ab	0.18 ± 0.03 b	88.8 ± 15.3 b
Sat-4	13,449 ± 1142 c	906 ± 10.1 a	12.3 ± 0.48 ab	0.35 ± 0.29 a	27.0 ± 9.52 a	0.35 ± 0.40 b	0.20 ± 0.01 b	1.68 ± 0.38 a
**W-B**								
Sat-1	2486 ± 2081 a	382 ± 103 a	17.4 ± 5.54 a	185 ± 114 a	0.93 ± 0.15 a	185 ± 161 a	0.04 ± 0.01 a	0.10 ± 0.03 a
Sat-2	5520 ± 1021 ab	727 ± 75.4 ab	8.75 ± 2.26 a	45.4 ± 33.0 a	1.07 ± 0.14 a	45.4 ± 46.7 ab	0.09 ± 0.02 ab	0.76 ± 0.21 a
Sat-3	10,924 ± 1398 ab	806 ± 59.8 ab	11.1 ± 1.45 a	1.19 ± 0.14 a	20.0 ± 11.3 a	1.19 ± 0.20 bc	0.15 ± 0.03 bc	1.04 ± 0.28 a
Sat-4	13,029 ± 1554 b	909 ± 58.1 b	0.60 ± 0.15 a	0.02 ± 0.00 a	33.7 ± 7.59 a	0.02 ± 0.00 c	0.19 ± 0.01 c	31.3 ± 10.6 b
**W-C**								
Sat-1	737 ± 306 a	312 ± 95.1 a	15.6 ± 4.47 b	105 ± 37.5 a	0.91 ± 0.26 a	105 ± 53.1 a	0.03 ± 0.01 a	0.08 ± 0.03 a
Sat-2	3061 ± 836 a	520 ± 96.0 ab	5.76 ± 1.30 ab	112 ± 0.58 a	1.21 ± 0.40 a	112 ± 0.82 a	0.09 ± 0.01 ab	0.84 ± 0.10 a
Sat-3	7253 ± 1089 ab	736 ± 101 ab	0.31 ± 0.16 a	499 ± 93.9 b	61.2 ± 11.3 b	499 ± 133 ab	0.14 ± 0.02 b	22.3 ± 7.91 c
Sat-4	13,731 ± 1825 b	939 ± 121 b	1.73 ± 0.00 a	0.09 ± 0.00 a	17.6 ± 4.21 a	0.09 ± 0.00 b	0.18 ± 0.03 b	9.03 ± 2.84 b

(**a**) O_max_: maximum/initial oxygen value (hPa); O_min_: minimum/residual oxygen value (hPa); ∆O_max_min_: total oxygen consumed (hPa); O_int_: oxygen value when half of the total consumption time has elapsed (hPa); O_90_: oxygen representing 90% of the range between the maximum and minimum values, i.e., when the first 10% of total oxygen (hPa) had been consumed; ∆O_90_10_: variation between 90% and 10% of oxygen (hPa); A_max_min_: area under the oxygen consumption curve (hPa); AO_max_min_: area under the oxygen consumption curve between maximum and minimum oxygen (hPa). For the same row, different letters indicate significifferences between the different saturation levels for W-A, W-B, and W-C, according to the Tukey’s test (*p* < 0.05). (**b**) ∆O_90_10_: variation between 90% and 10% of oxygen consumed (hPa); t_Omin_: time in hours that it took to consume all the oxygen (h); t_0_90_: time it took to reach O_90_ (h); t_R_min_: time it took to reach the minimum oxygen consumption rate (h); R_max_: rate of oxygen consumption (hPa/h); R_mean_: the area divided by the time it took to consume; V_cons_: rate of consumption of ∆O_max_min_ (hPa/h); V_cons10%_: rate of consumption to consume the first 10% of oxygen consumed (O_90_) (hPa/h). For the same row, different letters indicate significant differences between the different saturation levels for W-A, W-B, and W-C, according to the Tukey’s test (*p* < 0.05).

**Table 2:** Pearson correlation coefficients obtained between the consumption kinetics parameters and the difference between the chemical parameters obtained for W-A, W-B, and W-C after each saturation situation compared to the samples kept inert (* *p* level < 0.05, ** *p* level < 0.01, *** *p* level < 0.001).

	O_max_			O_min_			∆O_max_min_			R_max_		
	W-A	W-B	W-C	W-A	W-B	W-C	W-A	W-B	W-C	W-A	W-B	W-C
Df-3-Gl (Df)	−0.947 ***	−0.895 **	−0.732 *	−0.626	−0.616	−0.659	−0.922 ***	−0.900 **	−0.679	−0.695	−0.718 *	−0.681
Cy-3-Gl (Cn)	0.382	−0.492	−0.840 **	0.272	−0.503	−0.538	0.361	−0.471	−0.822 *	0.477	−0.626	−0.581
Pt-3-Gl (Pt)	0.407	−0.829 *	−0.787 *	0.469	−0.139	−0.206	0.377	−0.836 **	−0.809 *	0.426	−0.752 *	−0.462
Pn-3-Gl (Pn)	−0.125	−0.891 **	−0.413	0.129	−0.433	−0.269	−0.085	−0.895 **	−0.397	−0.091	−0.720 *	−0.338
Mv-3-Gl (Mv)	−0.932 ***	−0.800 *	−0.962 ***	−0.406	−0.291	−0.484	−0.908 **	−0.819 *	−0.950 ***	−0.553	−0.582	−0.595
% ABTS	−0.888 **	−0.552	−0.621	−0.568	−0.399	−0.462	−0.867 **	−0.574	−0.556	−0.754 *	−0.323	−0.666
% DPPH	−0.883 **	−0.761 *	−0.102	−0.539	−0.538	−0.313	−0.853 **	−0.786 *	−0.061	−0.729 *	−0.536	−0.225
TPI	0.953 ***	0.784 *	0.330	0.498	0.483	0.280	0.932 ***	0.791 *	0.312	0.674	0.549	0.000
Color Intensity (CI)	0.306	−0.570	−0.024	0.015	−0.357	0.364	0.332	−0.586	−0.119	0.305	−0.331	0.705
L*	−0.033	0.585	−0.418	0.134	0.350	−0.584	−0.067	0.599	−0.326	−0.095	0.367	−0.872 **
a*	0.035	0.607	−0.861 **	0.165	0.377	−0.601	0.000	0.622	−0.813 *	−0.048	0.397	−0.765 *
b*	−0.032	0.594	−0.672	0.143	0.368	−0.559	−0.066	0.608	−0.630	−0.086	0.380	−0.420
Cab*	0.005	0.599	−0.869 **	0.152	0.368	−0.614	−0.030	0.613	−0.820 *	−0.067	0.387	−0.753 *
650 nm	−0.086	−0.625	0.816 *	−0.193	−0.341	0.628	−0.050	−0.638	0.757 *	0.003	−0.434	0.808 *
630 nm	0.113	−0.566	0.718 *	−0.121	−0.361	0.630	0.147	−0.578	0.647	0.122	−0.350	0.873 **
620 nm	0.183	−0.546	0.612	−0.088	−0.335	0.621	0.217	−0.562	0.533	0.169	−0.318	0.900 **
520 nm	0.174	−0.658	−0.574	−0.024	−0.378	−0.022	0.200	−0.672	−0.640	0.262	−0.424	0.172
420 nm	0.566	−0.262	0.457	0.117	−0.264	0.546	0.588	−0.279	0.373	0.407	−0.021	0.923 ***
330 nm	0.685	0.291	0.092	0.122	−0.041	0.396	0.711 *	0.285	0.012	0.374	0.468	0.854 **

## Data Availability

The data are available upon request to the authors.
